# Animal Cruelty and Neighborhood Conditions

**DOI:** 10.3390/ani10112095

**Published:** 2020-11-11

**Authors:** Laura A. Reese, Joshua J. Vertalka, Cassie Richard

**Affiliations:** 1 Urban and Regional Planning and Political Science, Michigan State University, 517-353-5942, Human Ecology Building 552W, Circle Drive, Room 208A, East Lansing, MI 48824, USA; 2 Lucid Spaces, 200 N, Grand Ave, Lansing, MI 48933, USA; joshua.j.vertalka@gmail.com; 3 Oregon Commission for the Blind, 535 SE 12th Ave, Portland, OR 97214, USA; cassier140@gmail.com

**Keywords:** animal cruelty, neighborhood disorganization, theories of animal cruelty

## Abstract

**Simple Summary:**

Animal cruelty appears to be widespread. Competing theories have been posed regarding the causes of animal cruelty leading to conflicting findings and little direction for public policies to combat it. Using data from police department reports of animal cruelty in the City of Detroit from 2007 to 2015 this project assesses competing theories of the causes of animal cruelty. The findings suggest that deviance and social disorganization theories best account for animal cruelty. Neighborhood conditions in terms of economic stress, vacancy and blight, and crime appear to have the greatest impact on animal cruelty in this urban area.

**Abstract:**

Background: Animal cruelty appears to be widespread. Competing theories have been posed regarding the causes of animal cruelty leading to conflicting findings and little direction for public policies to combat it. Objective: To assess the applicability of extant theories of the causes of animal cruelty: domestic violence; deviance; perpetrator traits; and social disorganization. Methods: Data are drawn from police department reports of animal cruelty in the City of Detroit from 2007 to 2015; 302 incidences of animal cruelty were reported. Multiple regression is used to determine the theory which best appears to account for animal cruelty. Results: Common types of animal cruelty in Detroit are shooting; blunt force trauma; neglect; and dogfighting. While most incidents involve unknown persons; cruelty by owners; neighbors; and domestic partners is also common. Neighborhood conditions in terms of economic stress; vacancy and blight; and crime appear to have the greatest impact on animal cruelty. Conclusions: The findings from Detroit support deviance and social disorganization theories of animal cruelty. Neighborhood conditions in terms of economic stress, vacancy and blight, and crime appear to have the greatest impact on animal cruelty in this urban area.

## 1. Animal Cruelty and Neighborhood Conditions

Research on animal cruelty has grown exponentially and across disciplines over the course of the last two decades although work focusing explicitly on urban areas is relatively rare. Additionally, because of the complexity of the issue, it has been noted that there remains a limited understanding of companion animal cruelty:

“Companion animal cruelty is a surprisingly complex phenomenon, involving a multitude of different situational factors, motives, and other potential causes…there is no single type of companion animal cruelty offense, nor is there one typical type of companion animal cruelty offender.” [1]

It appears that animal cruelty is quite widespread. Although based on a sample of college students, recent research has suggested that 55% of surveyed respondents had intentionally harmed or killed at least one animal [2]. Because the inclusion of animal cruelty in the FBI database is recent (2016) and reporting voluntary, it is difficult to calculate national rates of cruelty in the US but it has been suggested that the available figures are underestimated because so much cruelty is hidden and otherwise unreported [3]. A survey conducted in Australia indicated that animal mistreatment was common with almost 26% of respondents reporting that they had witnessed mistreatment although the most typical perception was that they were seeing neglect as opposed to more active forms of cruelty [4].

Research across countries has attempted to assess the extent of different types of animal cruelty, although comparable data are consistently problematic. Carlisle-Frank and colleagues investigated the animal cruelty of abusers as reported by women in domestic violence shelters [5]. Forty-three percent of respondents indicated that their significant other had punched, hit, choked, drowned, shot, stabbed, or thrown their animals against walls or down stairs. Denial of food, water, and veterinary care occurred in 26% of the cases. The most common forms of animal cruelty in a South African study were the restriction of movement, insufficient food or water, abandonment, neglect, lack of veterinary care, and assault [6]. A study conducted in Australia found that the most common complaints of animal cruelty, particularly for puppies, involved neglect such as poor body weight, lack of food and water, and being confined in such a way that the animal lacked appropriate exercise. Adult dogs were exposed to more active forms of cruelty such as poisoning [7]. The most frequently reported forms of animal cruelty in Manitoba, Canada, are also related to neglect (lack of food and water) and most commonly involve dogs. In Winnipeg, Canada, issues most often reported include animals locked in cars, left in conditions of extreme heat or cold, insufficient food and water, and abandonment. However, research on cruelty enforcement has found that prosecution is rare, occurring in only the most serious cases [8].

There have been calls in the academic literature to examine animal cruelty in light of larger social factors and relationships to address the complexity of causes [9]. This study does this by looking at a broadly-based set of potential correlates of animal cruelty in an urban setting including characteristics of the perpetrator, relationship between the abuser and the nonhuman animal victim, the neighborhood environment where the cruelty took place, and the types of cruelty that occurred. In doing so it weighs the applicability of four theories or explanations for animal cruelty in an urban setting highlighting the complexity of the issue and setting directions for public policies that acknowledge and address those complexities.

The article proceeds by discussing the major strands of theory and research exploring the causes and correlates of animal cruelty: human relationships/domestic violence, deviance/criminality, perpetrator traits, and social disorganization. Gaps in extant knowledge are summarized and the current research positioned within those gaps. To provide an explicit urban focus, the City of Detroit is used as the case for the analysis of police reports of animal cruelty to follow. Explanatory models are developed and tested and the implications for urban public policy addressed.

### 1.1. Animal Cruelty and Human Relationships/Domestic Violence

The relationship between animal cruelty and domestic violence has been extensively studied [10]. Forty-seven to seventy one percent of women in domestic violence shelters indicated that their partners had abused or threatened their pets [11]. Research comparing women that had and had not been abused found that cruelty to pets was reported by 53% of the former but only 13% of the latter [12]. Forty-one percent of men arrested for domestic violence reported committing animal cruelty as adults; the rate of animal cruelty in the general population is 1.5% [13]. Animal cruelty by domestic partners and other family members was more prevalent in violent families compared to those without domestic violence [10]. A Canadian study indicated that women reporting violence to their animals tended to suffer abuse themselves with greater frequency and severity [14].

However, recent research has suggested that domestic partners are not the only source of animal cruelty resulting from interpersonal human relationships. Animal cruelty is part of a complex of aggression that includes assault against humans, domestic violence, intimidation, and harassment. While much research has focused on romantic partners, family members and neighbors are also involved in animal cruelty [15]. Perpetrators that assault humans are also more likely to use direct force against animals. Individuals that shoot other people are more likely to shoot animals. Thus, the same modes of violence play out against both human and nonhuman animals [16].

### 1.2. Animal Cruelty: Deviance and Criminality

Animal cruelty has been framed as an indicator of antisocial behavior in children potentially leading to violence in adulthood [17]. This “link” or graduation hypothesis suggests that young violent offenders test their abusive skills on animals and take this knowledge, and the desensitization that goes with it, on to humans [18]. A good bit of research has supported a link between being exposed to and engaging in animal cruelty as a child and perpetrating animal cruelty and violence against humans as an adult [19]. Yet, this research has been limited to small and unrepresentative samples and work with larger datasets has not found the graduation hypothesis to be supported [20]. As a result other scholars have proposed a generalized deviance theory to explain animal cruelty suggesting that it is just one type of behavior that is tied to a complex of others such as drug use and criminality [21].

Partly tied to, and supporting, deviance theory of animal cruelty, research has explored the connections between animal cruelty and other types of crime. For example, dog fighting is related to other criminal acts such as drug and weapons offenses, and gang participation and involves dogs that are owned by the abuser [15]. Thus, it has been suggested that persistent poverty and lack of opportunities feed into a culture of gang activities which normalizes dog fighting [22].

It appears that a complex of individual traits and general criminal activity are related to animal abuse. For example, gender, race, and age were found to be correlated with animal crime in Chicago as were a juvenile crime record and crimes involving drugs and weapons leading to the conclusion that animal abusers are more similar to common street criminals than both the graduation hypotheses or studies of pathologies would suggest [3]. Thus, animal abuse is tied to both violence generally and other types of crime, including violent crime [23].

### 1.3. Animal Cruelty and Individual Traits of Perpetrators

Agnew posed a social psychological model of the causes of animal abuse with “social position” (gender, age, race, education) along with individual traits such as degree of empathy, self-control, and ignorance about the consequences of abusive behavior leading to animal cruelty [24]. A good bit of research has identified connections between gender, age, and animal cruelty. Overwhelmingly males are the more likely perpetrators, particularly for active and more violent forms of cruelty [1]. When women are involved in cruelty it is more likely to be passive such as neglect, hoarding, or involving poison [25]. The lower propensity for animal cruelty among women has been posited to emanate from their higher levels of empathy across animal types—pets, pests, and food—and their tendency to view themselves as animal guardians [26]. Most cruelty offenders have been found to be around 30 years old or younger although older individuals are more likely to neglect or hoard animals even in the presence of strong attachments to them [26].

Relationships between race, ethnicity, and animal cruelty have not been definitively determined in extant research, however, because of conflicting results. On the one hand, some work suggests that African Americans and Hispanics are the most frequent perpetrators of animal cruelty although racial segregation not race per se may be the driver [3]. However, other research has found white animal abuse offenders to be more common [27], that Hispanic pet owners have human-animal bonds similar to non-Hispanic whites [28], and that white partners are more likely than Hispanic ones to harm pets as part of domestic violence situations [29]. Further, whites have been found to be more engaged in dog fighting than individuals of color [30] while other research has suggested that the practice crosses racial and socioeconomic lines [31].

### 1.4. Animal Cruelty and Neighborhood Disorganization

A relatively recent body of research has begun to explore the larger environment of animal cruelty, specifically looking at potential connections between neighborhood conditions and rates of cruelty. Social disorganization theory posits that community structural disadvantages in terms of poverty and lack of education and opportunities, weaken social ties, social controls, and consensus against crime [32]. It has been suggested that animal cruelty will be higher in disorganized areas for these reasons [3]. For example, spatial analysis was used to identify correlations between neighborhoods with high levels of animal cruelty (including dog fighting) and domestic violence, child abuse, crime, Hispanic populations, and abandoned properties [33]. In a suburban setting, block groups with higher levels of social disorganization (measured as female-headed households, percent below the poverty level, unemployment, economic disadvantage, ethnic heterogeneity, housing tenure instability, and divorced and separated persons) were found to have more reported animal cruelty offenses [34]. General “community hardship” such as crowded housing, poverty, low income, percent of residents without a high school diploma, crime, and dependent children and seniors appears related to animal crimes [3]. Such research essentially shifts the unit of analysis from the psychological motivations of the individual perpetrator to neighborhood characteristics. However, by employing demographic indicators to measure “disorganization” such studies also run the risk of “blaming the victims” whereby systemic inequalities are redefined as neighborhood problems as opposed to conditions that place both human and nonhuman animals at risk.

### 1.5. Proposing and Testing a more Comprehensive Model

The research just discussed explores a variety of correlates of animal cruelty at different scales. Yet more work has been called for, particularly on community features that relate to, promote, or both, animal cruelty [34]. This research responds to that call by examining a broader array of potential correlates of animal cruelty at the sub-city level in an urban setting. Independent variables are drawn from across the literature just cited and include four categories of factors: traits of the perpetrators, relationships between perpetrator and animal including domestic violence, and socio-economic and physical neighborhood characteristics. Thus, by examining correlates across academic disciplines and approaches, the research provides a comprehensive examination of the factors related to animal cruelty in a major city.

## 2. Methods

This research focuses on animal cruelty in the City of Detroit because economic distress, vacancy and abandonment, high numbers of stray and feral dogs, high risk of dog bites, and dog fighting create circumstances ripe for animal cruelty [15]. Detroit’s economic distress limits its ability to provide animal welfare services including combating animal cruelty [35]. However, it should be noted that Detroit Animal Care and Control (the municipal agency responsible for animal control and welfare) has improved enforcement of animal welfare ordinances since the period of this study.

### Data and Variables

A variety of methods have been used to explore animal cruelty. Studies of domestic violence have typically involved small samples, recall interviews, and surveys with victims and perpetrators and with college students [2]. Research on incarcerated individuals, serial killers, and sadists also provides few cases and unique populations [36]. Broader studies have examined news reports of animal cruelty or used open source websites that include convictions for animal cruelty. These have limitations in that only the most serious or attention garnering cases are included, many cases are not in the public domain, or both [1]. Finally, other studies have employed police data but have not coded the text of police reports in detail thus missing information about the circumstances surrounding the incident, victim and perpetrator, the nature and conditions of the animals, and the demographic and locational context where the incidents occurred [34]. There have been specific calls for research that considers a broad range of potential factors involved in animal cruelty that incorporates administrative data such as police reports [1]. This research addresses these calls by using animal cruelty police data with the text describing each incident coded in detail.

Police reports: All animal cruelty incidents in Detroit between 2007 and 2015 for which there is a police report were included in the analysis. Animal cruelty was categorized into eight types of abuse: dog fighting, shooting, neglect, poisoning, threat, stabbing, kicking/hitting with blunt force, and other (see [15] for a full explanation of data and coding). Attributes of the suspect, relationships between the suspect and the owner of the dog (neighbor, family member, romantic partner), whether domestic violence was suspected, and circumstances surrounding the cruelty (type of animal, breed of dog, location where abuse occurred) were also coded. Number of cruelty reports by zip code serves as the dependent variable for the study (It would have been optimal to use a smaller unit of geography such as a census tract but there were too few reported incidents in many tracts for this to have been viable. There are 29 zip codes in the City of Detroit).

Neighborhood variables: The environmental variables represent factors that have been used in previous research to indicate neighborhood conditions. However, there are additional attributes of neighborhoods, not explored in the extant literature that might create environments ripe for animal cruelty. These include features that might attract roaming dogs and present hidden locations where dog fighting could occur (vacant buildings, blighted areas, parks and greenspaces, for example). Conversely, areas where adults and children congregate might lessen the likelihood of cruelty because many “eyes” are on the scene and they represent locales of routine activities; schools and bus stops, for example (see [37] for a detailed description of data and coding). The presence of liquor stores was also measured as they could represent a source of neighborhood disorder (increasing cruelty) or more active commercial activity (lowering cruelty).

Finally, to provide a sense of other potential demographic correlates, data for the zip code where the cruelty incident occurred were collected including: unemployment, percent below the poverty level, female-headed households below the poverty level, education, median rent, food stamp use, age structure, per capita income, and occupied and vacant housing. These variables assess the level of neighborhood poverty, economic stress, and opportunity structures that have been hypothesized to increase the risk of animal cruelty [37,38]. These data have been drawn from the 2011 American Community Survey estimates since that is roughly the midpoint of the range of the animal cruelty data.

## 3. Results

### 3.1. Nature of Cruelty Incidents

The dataset includes 302 police reports of animal cruelty over a period of nine years. It is likely that this represents an undercount of actual cruelty since reports may have gone to and been investigated by Detroit Animal Care and Control or the Michigan Humane Society also operating in Detroit. The most frequent types of animal cruelty in Detroit were shooting (23% of incidents), kicking/blunt force (21%), other (17%), neglect (12%), and dogfighting (10%). Stabbing (6%), poisoning (5%), and threatening to harm an animal (2%) are much less common. Thus, cruelty in Detroit appears different than in other studies indicating that neglect—restriction of movement, lack of food and water, abandonment, and lack of veterinary care—is the most common form of cruelty.

### 3.2. Traits of the Perpetrator

The perpetrators tend to be male (83%). Of the 150 suspects whose race is included in the police report, 89.3% are of color (It should be noted that 83% of Detroit residents are African American). Fewer than one third of the perpetrators (32%) were arrested for the animal cruelty incident.

Table 1 presents the results of the traits of the suspect as drawn from the police reports regressed on number of cruelty incidents in a zip code. The traits of the cruelty suspects overall do a very poor job of predicting cruelty; none of the individual variables are significantly correlated with cruelty in multiple regression.

### 3.3. Human Relationships/Circumstances Surrounding Cruelty

Thirty-six percent of the cruelty incidents were committed by an unknown person. The next mostly likely perpetrators were the owner of the animal (20%), neighbors of the owner (14%), domestic partners or others involved in a romantic relationship (It would have been ideal to have also measured cruelty committed by ex-partners of the human guardian of the animal but that information was not included in the police reports) (12%), or a family member (9%). The majority of the cruelty cases involved dogs (89%, 7% involved cats, and 2% other animals). This comports with other research which has indicated dogs as the most common victims of animal cruelty [1]. Among incidents involving a dog for which the breed is identified, 57% involved pit bulls (Detroit has not conducted a dog census to determine predominant types of dogs in the city nor is there an accessible data set on dog licenses. However, over 90% of the dogs in the city’s municipal animal shelter (Detroit Animal Care and Control) at any given time are likely some type of pit bull mix suggesting that there are high rates of pit bull ownership in the city [35]. The implications of this overrepresentation would be worthy of future study.). Of the cases involving dogs, 20% died as a result of their abuse. The preponderance of incidents occurred in the animal owner’s yard or home.

As a group, the variables measuring the relationship between the suspect and the animal victim do a slightly better job of accounting for variation in total incidents in a zip code than traits of the suspect (Table 2). The equation including attributes of the animal and relationship between animal and suspect accounts for 17% of the variation in cruelty. However, only one variable comes close to statistical significance; whether the owner was the suspect. Situations where domestic violence was noted in the police report as being present are not significantly correlated with number of cruelty incidents.

### 3.4. Socio-Economic Aspects of Neighborhood Disorganization

The socio-economic traits of the zip code where the cruelty occurred do a very good job of predicting the number of incidents of cruelty; 81% of the variation in cruelty is accounted for by the variables in the equation in Table 3. Zip codes that are more distressed in terms of unemployment and higher rents as a percentage of income have more reported cruelty incidents. Areas with more children under 5 years of age also have more cruelty.

### 3.5. Physical Aspects of Neighborhood Disorganization

Physical traits of the environment where the cruelty took place account for 72% of the variation in cruelty making it just slightly less robust than the forgoing socio-economic model (Table 4). Overall cruelty appears to be strongly related to neighborhood conditions: higher building vacancy and blight, more crime as represented by murders, fewer building permits, and lack of green spaces for recreation.

### 3.6. Combined Model

A final regression was run including only the variables most strongly related to the number of cruelty incidents in a zip code in the previous regression analyses. Results of the best fitting model are presented in Table 5. This model including environmental variables and whether the owner was the suspect accounts for 72% of the variation in animal cruelty (The demographic variables of rent to income, unemployment, and children under five were removed from the model due to high VIF. Cruelty by neighbor serves as the referent category for cruelty by owner). Cruelty is higher in areas with higher levels of blight, murder, a lack of building permits indicating low development activity, and less green space amenities.

## 4. Discussion and Policy Implications

Detroit has a different profile of animal cruelty than has been found in previous research; neglect is relatively less frequent than active forms of abuse. This may be due to high levels of criminal violence in the city generally or to the severity of the city’s economic decline. Exploring the factors causing more active cruelty in Detroit is something that should be considered in future research.

The findings from Detroit generally appear to support the social disorganization, and to a lesser extent the deviance/criminality, theories of animal cruelty. The only human relationship that remains significantly correlated to cruelty in multiple regressions is that of an owner. None of the traits of the accused—race, gender, or age—remain related to animal cruelty in multiple regression. In short, neighborhood conditions in terms of economic stress, vacancy and blight, and crime appear to have the greatest impact on animal cruelty in this urban area. In addition to lending support for the disorder theory the findings also speak to potential policy actions to combat animal cruelty.

That animal cruelty is tied to general patterns of deviance and other violent crime has led scholars and health professionals to recommend that it be used as a marker for other types of child and family abuse. The One Health movement has called for better communication and cross-reporting between the veterinary, social work, animal sheltering, and human medical communities in an attempt to reduce violence among all family members, both human and nonhuman animals [39]. It should be noted that there are recommendations that such cross-reporting be encouraged and facilitated but not made mandatory as it could create a disincentive for taking animals for medical care or not being open about abuse in the home due to fear of retaliation [17].

There are a variety of actions that might be taken to improve the neighborhood conditions that appear to be the most important correlates of animal cruelty. In a larger sense efforts to reduce economic stress and structural inequality in terms of unemployment and high rent burdens may decrease anxiety among pet owners, allow them to more easily afford pet food and medical care, and lessen the need to embrace dog fighting as a form of income production (from both gambling and the breeding and sale of dogs). Animal welfare advocates and organizations have increasingly begun to recognize inherent discrimination related to judgments about pet-keeping and enforcement of animal welfare and control ordinances [40]. Strict enforcement of such legislation often targets low income, minority, and immigrant communities [41]. As a result, a number of animal welfare organizations around the US have been implementing programs to support those struggling financially to keep their pets in their homes providing food, low cost medical care, training assistance, fencing, crates, and so on [42]. The American Society for the Prevention of Cruelty to Animals’ (ASPCA) Keeping Pets and People Together position statement underlies such efforts [43]. These programs recognize that in situations of neglect or passive cruelty specifically, economic deprivation is the driver and that increased enforcement is not the most appropriate response particularly since the concept of “neglect” of an animal is socially determined based on the “myth” of the irresponsible owner [42]. The partnership between the ASCPA and the New York Police Department (NYPD) to fight animal cruelty is an example of an effort to address these concerns. Here the resources of the NYPD are brought to bear on animal cruelty cases while the ASPCA focuses on the health and welfare of the nonhuman animal victims and also the needs of guardians in cases where pets may be able, with a supportive response, to remain in their homes [44].

However, other attributes of the environment are also related to cruelty. Addressing abandonment and blight should also reduce cruelty. Vacancy and blight are markers that a neighborhood has been left behind economically and socially. Many animals have been left in abandoned homes as the result of foreclosure and precarious housing options that often restrict size or type (read pit bull) of dog. Vacant structures provide places for stray and feral animals to hide. Checking vacant homes for animals, placing them in an animal shelter, and boarding up the structures or razing them entirely should reduce the cruelty that un-homed animals may experience on the streets and improve general welfare. Expanding programs to remove abandoned buildings and clear blighted lots should reduce habitats for stray and feral animals but also make the area more desirable and less open to crime and further deterioration. Neighborhoods with high vacancy and blight may suffer from other markers of economic distress thus endangering the social capital necessary for protective behaviors.

Finally, dog fighting in particular is tied to other types of criminality such as drug offenses and involves dogs owned by the perpetrator [15]. Crackdowns on dog fighting and breeding and drug and weapons possession and sales would likely reduce this type of animal cruelty. Cooperation and communication between public safety and humane organizations would help identify the locations of dog fighting based on the condition of animals ending up at animal control and would enhance police resources with investigators trained and experienced in animal cruelty investigations [3]. However, it should be noted that police and animal control responses to dog fighting and breeding are tied to social constructions of gender and race and increased enforcement efforts will likely fall more heavily on communities of color.

### Limitations

There are several limitations to this study which speak for needed future research. First, it would have been ideal to have explored neighborhood variation using a smaller geography than zip codes. Future research could collect data from a longer time period and from more cities to allow for sufficient cases of animal cruelty for census tract analysis. Second, the study is based in a single US city and thus findings might not be generalizable to other urban contexts. Third, the text of the police reports varied in level of detail and individual officers could have had different perceptions of a variety of variables such as breed of dog, severity of incident, and whether domestic violence was involved. Fourth, communities with more police are likely to evidence more police reports of animal cruelty because of greater resources, making comparisons across cities problematic. Further, there has been no research to date that explores the connections between different types of cruelty and the theories explored. For example, are the types of cruelty related to neighborhood conditions different from those that stem from domestic violence? There are reasons to suspect that this might be the case with domestic violence related to more violent cruelty such as beating and kicking animals [15] and animal abandonment potentially more likely in areas with greater housing vacancy. Testing these possibilities is ripe for future research. Finally, the analysis does not interrogate the nexus between the need to implement measures to identify and reduce animal cruelty and over policing in minority and immigrant communities which places their animals and themselves at greater risk of enforcement. The optimal balance between these realities is a necessary topic for future research.

## 5. Conclusions

While this research points to neighborhood conditions as the strongest predictors of animal cruelty, domestic violence and other interpersonal relationships, and in some cases, traits of the perpetrator, have been found to be related to different aspects of animal cruelty in other research. Thus, it is unlikely that solely focusing on conditions within the environment will address all aspects of animal cruelty. Systemic inequality breeds many conditions that put both humans and animals at risk of violence and greatly challenges the ability of even caring animal guardians to meet the needs of their pets. Better understanding the complexity of animal abuse can only lead to more effective and multi-pronged efforts to combat it.

## Figures and Tables

**Table 1:** Regression of traits of perpetrator (Female serves as the referent category for gender. Age of perpetrator was collapsed into ordinal categories; there were insufficient incidents for child and adult suspects to calculate regression data) and animal cruelty.

Variables	Estimate	Std.Error	*t* Value	Probability	Variance Inflation Factor
Perp. White	−8.40	1.53	7.34	0.29	1.05
Perp. of color	0.87	1.64	0.53	0.60	4.46
Male	−1.85	1.91	−0.97	0.34	4.46
Teen	−9.26	7.65	−1.21	0.24	1.00
Constant	11.25	1.53	7.34	1.39	
Adjusted R^2^ = 0.02					

**Table 2:** Regression of human relationships/circumstances surrounding cruelty. (A number of variables were removed from the equation due to high VIFs including: location of cruelty; breed of dog other than pit bull designation; abuse caused by police, unknown persons, in the course of a break-in, and neighbors; whether there was a history of abuse or bites for the animal involved; type of animal involved; specific nature of the cruelty; and number of animals involved. Reference categories thus include cruelty not part of domestic violence, cruelty by neighbor, and other dog breeds).

Variables	Estimate	Std.Error	*t* Value	Probability	VIF
With domestic violence	−0.45	2.19	−0.21	0.84	3.73
Owner is suspect	2.33	1.23	1.89	0.07	2.19
Pit bull	−0.66	0.94	−0.70	0.49	1.10
Family member is suspect	1.64	0.94	0.70	0.49	2.58
Constant	7.18	1.70	4.24	0	
Adjusted R^2^ = 0.17					

**Table 3:** Regression of relationship between socioeconomics of the neighborhood and cruelty (Several demographic variables were removed from the equation due to high VIFs including: median household income, % with a bachelor’s degree, and % with a high school degree.).

Variables	Estimate	Std.Error	*t* Value	Probability	VIF
Rent/income	0.335	0.133	2.51	0.02	2.67
Unemployment	0.00172	0.000828	2.08	0.05	5.39
% in poverty	2.85	7.05	0.41	0.69	1.37
% kids under 5	0.00180	0.000914	1.97	0.06	3.39
Constant	−13.6	4.03	−3.38	0	
Adjusted R^2^ = 0.81					

**Table 4:** Regression of relationship between physical characteristics of the neighborhood and cruelty (Variables removed from the equation due to high VIF include: bus stops; robbery; assault; land bank properties; liquor stores; parks; childcares; and middle and elementary school buildings).

Variables	Estimate	Std.Error	*t* Value	Probability	VIF
Vacancy	0.0132	0.00532	2.48	0.02	2.95
Blight	0.206	0.101	2.05	0.05	1.24
Murder	0.00266	0.000980	2.71	0.01	3.12
Building Permits	−0.0254	0.0109	−2.33	0.03	1.31
Green spaces	−0.231	9.59	−2.41	0.02	1.21
Constant	−0.0725	2.11	−0.34	0.73	
Adjusted R^2^ = 0.72					

**Table 5:** Regression of best fitting model across categories of variables.

Variables	Estimate	Std.Error	*t* Value	Probability	VIF
Vacancy	9.01	6.12	1.47	0.15	4.03
Blight	2.03	9.92	2.05	0.05	1.24
Murder	2.97	9.94	2.99	0.01	3.31
Building Permits	−2.54	1.07	−2.27	0.03	1.31
Green spaces	−2.02	9.70	−2.08	0.05	1.28
Owner is suspect	8.00	6.05	1.32	0.20	1.60
Constant	−4.77	2.09	−0.23	0.82	
Adjusted R^2^ = 0.73					
